# Bridging the Gaps: Addressing Inequities in Neurological Care for Underserved Populations

**DOI:** 10.1111/ene.70073

**Published:** 2025-02-06

**Authors:** Olivier Uwishema, Paul Boon

**Affiliations:** ^1^ Department of Research and Education Oli Health Magazine Organization Kigali Rwanda; ^2^ Department of Neurology, 4Brain, Reference Center for Refractory Epilepsy Ghent University Hospital, and Ghent University Ghent Belgium

**Keywords:** Africa, LMICs, low‐ and middle‐income countries, neurological care inequities

## 1 Introduction

With over 3 billion individuals affected globally, disorders of the nervous system are now a major contributor to both economic burden and morbidity. The global burden of neurological disorders is estimated to be 43% according to the most recent Global Burden of Disease Study. Neurological disorders are the main drivers of disability‐adjusted life‐years and mortality in the non‐communicable disease category. In addition, according to the systematic review by Lanza et al. disparities in the management of neurological disorders exist, disproportionately affecting underprivileged groups [1, 2, 3] With the majority of research concentrating within high‐income countries (HICs) when compared to their counterpart low‐ and middle‐income countries (LMICs), this study elucidates glaring discrepancies based on socioeconomic position, geographic location, and structural impediment.

**FIGURE 1:**
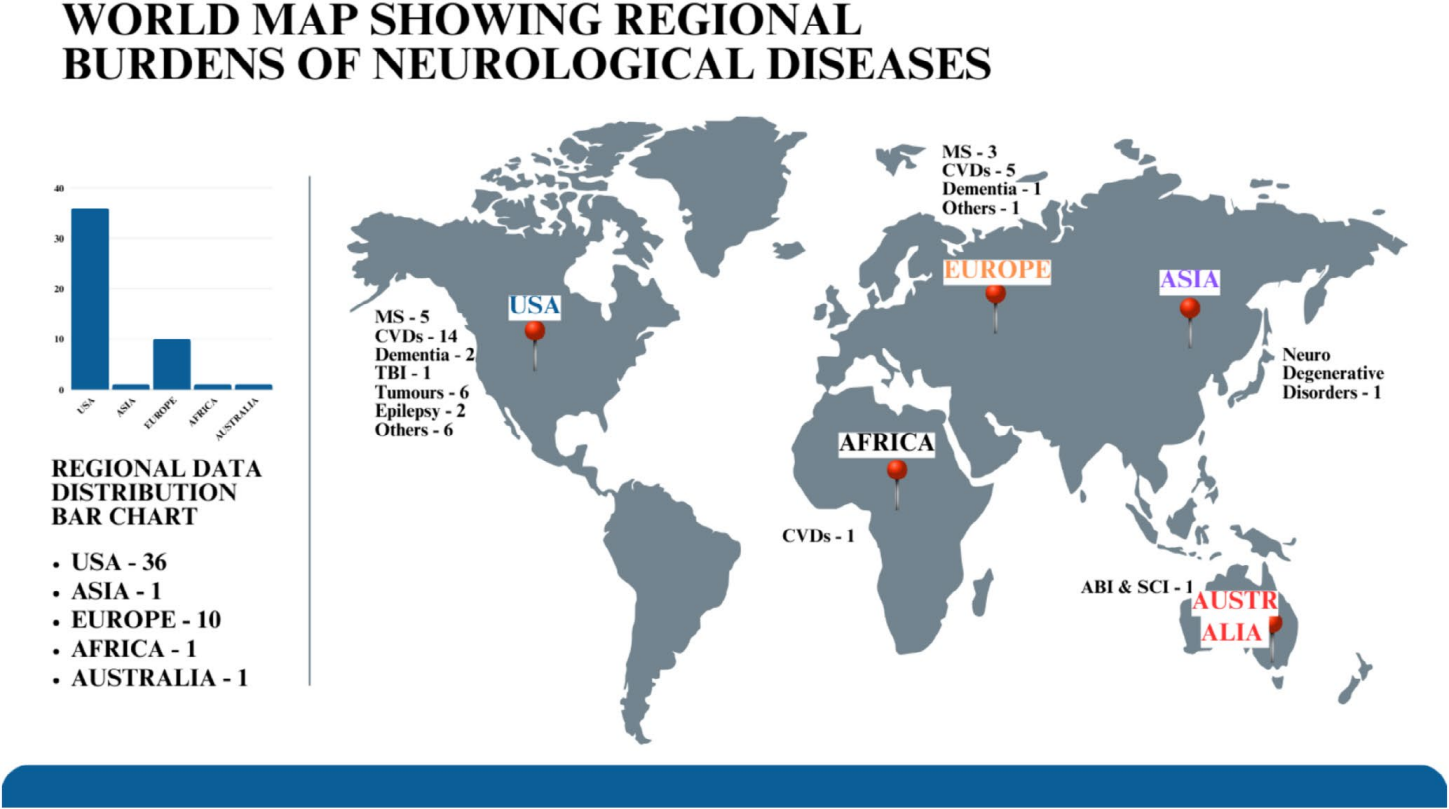
World map showing the regional burden of neurological disorders.

This oversight is conspicuously evident in Africa, where healthcare systems often contend with the dualistic burden of communicable and non‐communicable diseases [2]. Inequities circumventing healthcare surrounding neurological disorders, compounded by limited access and availability of resources, a dearth in primary clinical and research infrastructure, and a lack of trained medical and nursing personnel, remain a pressing but underexplored issue [3, 4]. The time to act is now. Addressing these inequities is not merely a public health imperative but a moral one, requiring dynamic global collaboration and context‐sensitive solutions. (See Figure 1).

## 2 Contextualization of Inequities in LMICs

While Lanza and colleagues (2024) meticulously documented inequities evident in the healthcare of neurological disorders worldwide [1], the study inadvertently mirrors the ever‐growing fissure in research it so critiques: the absence of robust data from LMICs. Of the 49 studies reviewed, only one was conducted in Africa [1]. This disparity is a testament to the systemic absence of LMICs from the global research agenda, perpetuating a vicious cycle of neglect.

**FIGURE 2:**
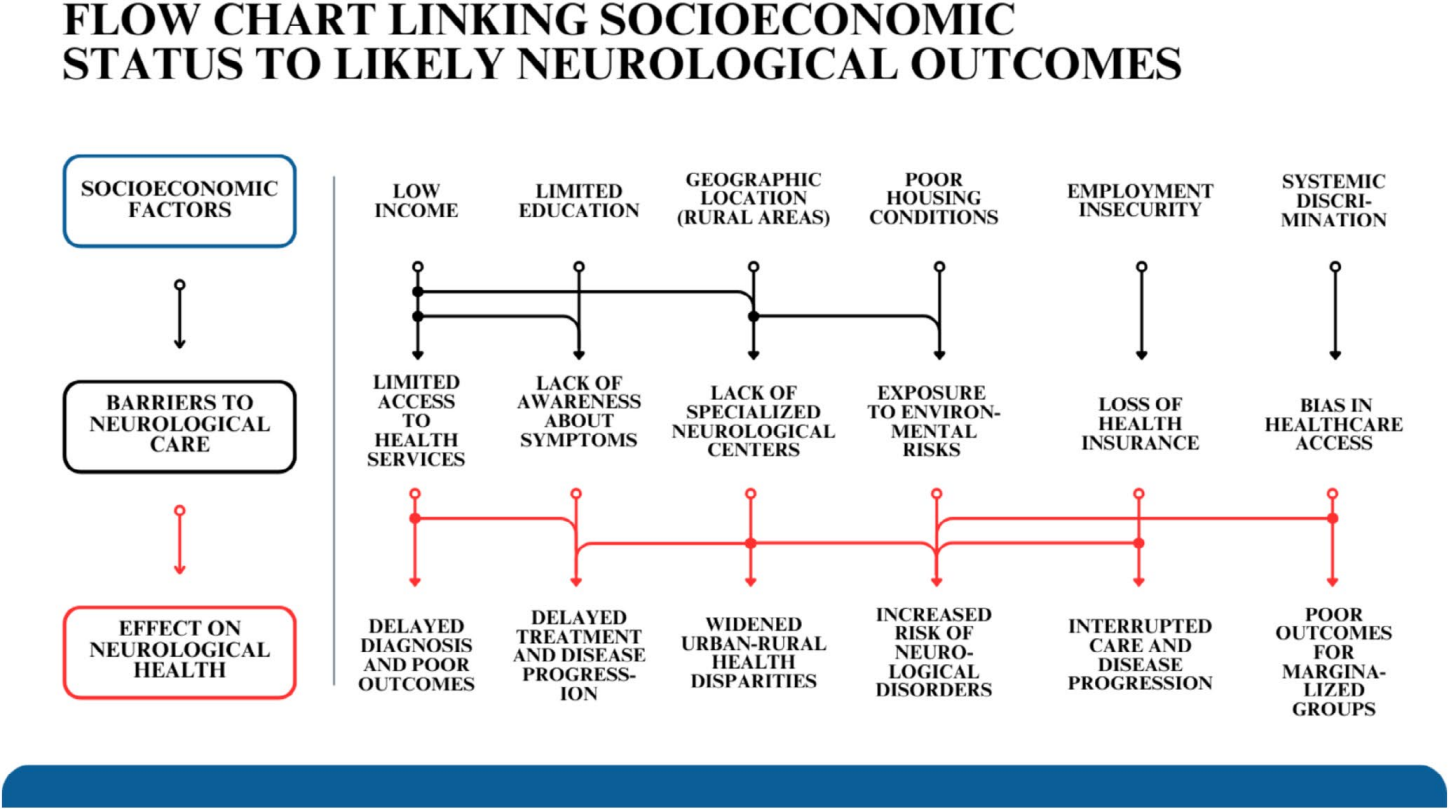
Flowchart linking socioeconomic status to likely neurological outcomes.

In LMICs, neurological care inequities are exacerbated by structural deficiencies. For example, patients living with epilepsy in rural Africa often rely on traditional healers due to sticking to traditional values and beliefs and, in addition, to the lack of neurologists and allied healthcare professionals, resulting in delayed or inadequate therapy [5]. In Guinea, a study found that 79% of epilepsy patients had consulted traditional healers, with 71% seeking their services before approaching medical providers, leading to delays in receiving appropriate treatment. Stroke survivors, disproportionately affected by comorbidities pertaining to hypertension and diabetes mellitus [3, 4, 6], face limited access to rehabilitation services [7]. These disparities affect not just access to care but also healthcare advocacy, data, and knowledge (See Figure 2).

## 3 Specific Challenges in Africa

### 3.1 Human Resource Deficits

Africa has fewer than one neurologist per million persons, compared to 15 per million in Europe [5]. This acute shortage, coupled with the brain drain phenomenon [8], leaves many patients without implicated specialization of healthcare necessitated.

### 3.2 Infrastructure and Equipment Gaps

Dedicated diagnostic tools comprising magnetic resonance imaging (MRI), computed tomography (CT) scanners, and electrophysiologic techniques (EEG, EMG, EP,…) are rare, and habitually confined to more urban institutions [4]. For rural populations, these services are not only inaccessible but also prohibitively expensive [7].

### 3.3 Financial Barriers

In many African domiciles, out‐of‐pocket expenses account for a significant portion of healthcare expenditures [9]. For low‐income families, a neurological diagnosis often spells financial ruin [8].

### 3.4 Gender, Cultural, and Educational Barriers

Women with neurological disorders (e.g., epilepsy) are generally more vulnerable and experience higher levels of inequity and stigma. In general, neurological disorders are associated with high levels of stigma (e.g., in epilepsy), which is associated with widespread reliance on traditional medicine and discourages patients from seeking expert healthcare [8, 10]. This is compounded by low levels of public awareness and knowledge regarding the management of neurological diseases, especially within the community [8].

## 4 Leveraging Global Health Initiatives

The World Health Organization Intersectoral Global Action Plan on Epilepsy and Other Neurological Disorders (WHO iGAP) offers a promising standardized framework to address these issues [2]. However, its global recommendations must be tailored to the specific realities of LMICs [11, 12, 13].

For instance, WHO iGAP advocates for capacity building through subsequent provision of education and training initiatives [2]. In Africa, this may translate to incentivizing local medical graduates to specialize in neurology through scholarships and programs adopting loan forgiveness [6]. Telemedicine, one of many WHO iGAP recommendations stipulated, may bridge the growing hiatus between rural patients and urban specialists [9], provided that governments invest in digital infrastructure and increased internet accessibility.

Furthermore, LMICs must be given priority in international financial arrangements. Strategies that have been effective in raising funds for communicable diseases, such as those of the Global Fund or Gavi, may be modified to assist with neurological care.

## 5 Recommendations

To bridge said gaps highlighted by Lanza et al. [1] we propose the following actions (See Table 1):

**TABLE 1:** shows the recommended strategies to address inequalities in neurological care.

Recommended strategy	Target group	Expected outcome
Improve education for healthcare professionals, planning specific training with mandatory updates.	Healthcare Professionals	Healthcare spaces with equitable disease management, free of racial, ethnic, gender or social stigma.
More research funding should be allocated to LMICs to fill the gap in information on health care.	Patients in LMICs	Local research‐driven neurological care and tailored interventions.
Geographical inequities can be minimized through the possibility of care through telemedicine.	Patients in Remote or Rural areas	Consistent access to healthcare services for individuals in underserved or rural regions.
Financial intervention aimed at reducing economic factors, such as improving financial support for disadvantaged groups.	Economically Disadvantaged Groups	Programs and paths that guarantee access to healthcare in disadvantaged groups.
Intervention can be aimed at increasing access to the internet and technological tools.	Technologically Disadvantaged Communities	Widespread connectivity providing access to telemedicine and other digital health resources.

### 5.1 Region‐Specific Research

African governments, academic institutions, and international organizations must prioritize research on neurological disorders. This includes establishing regional neurological registries to collate, analyze, and synthesize patient data garnered [4].

### 5.2 Investment in Medical Education

Expansion of neurology residency programs alongside the incorporation of specialist neurological training into primary healthcare curriculums should be fostered. Partnerships with HICs may facilitate knowledge exchange and expert mentorship [5].

### 5.3 Strengthening Infrastructure

Equip district hospitals with basic neurological diagnostic apparatus and rehabilitation facilities [7]. Public‐private partnerships could aid the subsidization of healthcare costs accrued.

### 5.4 Leveraging Technology

Promote telemedicine to connect rural communities with respective neurology specialists [9]. Mobile health applications remain already successful in HIV/AIDS care [8] and may display potential in its use for neurological disorders.

### 5.5 Community Engagement

Conduct awareness campaigns to reduce stigma and promote earlier diagnoses. Collaborate with local leaders and community health workers to build trust and disseminate information [10].

## 6 Conclusion

Neurological care inequities represent a microcosm of broader health disparities, reflecting systemic failures in access, funding, and prioritization. The findings of Lanza et al. provide a crucial starting point for dialog but must be complemented by action, particularly in LMICs like those in Africa.

Global collaboration is key. Academic journals, policymakers, and healthcare organizations must work together to amplify the voices of underserved populations. By prioritizing equity in neurological care, we may not only improve health outcomes but also uphold the fundamental principle of health as a human right.

The challenge is formidable, but the opportunity to transform lives is immeasurable. Bridging these gaps is not just an act of healthcare—it is an act of justice.

## Author Contributions

**Olivier Uwishema:** conceptualization, writing – original draft, investigation, methodology, visualization, writing – review and editing, formal analysis, project administration, resources, data curation. Table 1 and Figures were created by Olivier Uwishema. **Paul Boon:** writing – original draft, writing – review and editing, supervision, resources, formal analysis, methodology, data curation.

## Ethics Statement

The authors have nothing to report.

## Consent

The authors have nothing to report.

## Conflicts of Interest

The authors declare no conflicts of interest.

## Data Availability

The authors have nothing to report.
